# Intra- and Inter-Subunit Disulfide Bond Formation Is Nonessential in Adeno-Associated Viral Capsids

**DOI:** 10.1371/journal.pone.0032163

**Published:** 2012-02-28

**Authors:** Nagesh Pulicherla, Pradeep Kota, Nikolay V. Dokholyan, Aravind Asokan

**Affiliations:** 1 Gene Therapy Center, The University of North Carolina at Chapel Hill, Chapel Hill, North Carolina, United States of America; 2 Department of Genetics, The University of North Carolina at Chapel Hill, Chapel Hill, North Carolina, United States of America; 3 Department of Biochemistry and Biophysics, The University of North Carolina at Chapel Hill, Chapel Hill, North Carolina, United States of America; University of Kansas Medical Center, United States of America

## Abstract

The capsid proteins of adeno-associated viruses (AAV) have five conserved cysteine residues. Structural analysis of AAV serotype 2 reveals that Cys289 and Cys361 are located adjacent to each other within each monomer, while Cys230 and Cys394 are located on opposite edges of each subunit and juxtaposed at the pentamer interface. The Cys482 residue is located at the base of a surface loop within the trimer region. Although plausible based on molecular dynamics simulations, intra- or inter-subunit disulfides have not been observed in structural studies. In the current study, we generated a panel of Cys-to-Ser mutants to interrogate the potential for disulfide bond formation in AAV capsids. The C289S, C361S and C482S mutants were similar to wild type AAV with regard to titer and transduction efficiency. However, AAV capsid protein subunits with C230S or C394S mutations were prone to proteasomal degradation within the host cells. Proteasomal inhibition partially blocked degradation of mutant capsid proteins, but failed to rescue infectious virions. While these results suggest that the Cys230/394 pair is critical, a C394V mutant was found viable, but not the corresponding C230V mutant. Although the exact nature of the structural contribution(s) of Cys230 and Cys394 residues to AAV capsid formation remains to be determined, these results support the notion that disulfide bond formation within the Cys289/361 or Cys230/394 pair appears to be nonessential. These studies represent an important step towards understanding the role of inter-subunit interactions that drive AAV capsid assembly.

## Introduction

Adeno-associated viruses (AAV) are members of the parvovirus family with a spherical T = 1 capsid packaging a 4.7 kb single-stranded DNA genome [1]. The capsids are composed of total 60 copies of the viral protein subunits VP1, VP2 and VP3 in the ratio 1∶1∶10. The VPs are generated from overlapping reading frames and interact with each other through common VP3 subunit regions at the icosahedral two-fold, three-fold and five-fold axes of symmetry [2]. The critical role of interfacial amino acid residues within capsid subunit trimers and pentamers in parvoviral assembly is well known [2]. For instance, seven evolutionarily conserved amino acids were found essential for self-assembly of capsid subunits in the Minute Virus of Mice (MVM) [3]. These residues either participate in masking large hydrophobic surfaces on subunit association or form buried hydrogen bonds and salt bridges along a thin equatorial belt surrounding each trimer. Further, the assembly of MVM capsids has been shown to be mediated through assembly of such trimeric intermediates [4], [5].

In case of AAV capsids, the residues lining the two-fold (dimer) and five-fold (pentamer) interfaces are highly conserved between serotypes, while those creating the three-fold topology are more extensive and variable [6], [7]. The three-fold interface mostly involves interactions among residues located in the loop between the βG and βH strands. Trimer formation involves interactions between amino acids in as many as six variable regions within this GH loop. Residues within the eight-stranded β-barrel domain and the conserved α-helical domains form the majority of the five-fold and two-fold interface contacts. In addition to structural analysis, the latter observations have been validated by mutagenesis studies, which have helped identify key amino acids that play a critical role in AAV capsid assembly. For instance, alanine scanning mutagenesis of charged amino acid residues in the AAV2 VP1 subunit yielded several mutants that were defective in capsid assembly [8]. Most mutations discovered in the latter study were located within or near β-strands in the VP3 region. In addition, reduced VP expression and/or impaired capsid assembly has also been observed in case of five-fold pore mutants as well as mutants with deleted or truncated HI loop domains flanking the pore region [9]–[11].

The current study is focused on dissecting the role of cysteine residues and disulfide bond formation in AAV capsids. The VP subunit contains five highly conserved cysteines, which remain mostly buried within the capsid. No evidence indicating the formation of disulfide bonds has been reported in the structural studies of AAV capsids [6], [7], [12], [13]. However, it is conceivable that disulfides can potentially be formed due to the observed proximity between certain cysteine residues in the VP subunit. Using a panel of capsid subunit mutants, we demonstrate that none of the five cysteine residues appear to be involved in disulfide bond formation. Interestingly, the VP subunits of mutants C230S and C394S are rapidly degraded within host cells. Further, while the C230V mutant is not viable, the C394V mutant forms infectious particles similar to wild type (wt) AAV2 capsids. Thus, the thiol side chain of Cys394 appears dispensable and consequently disulfide bond formation between Cys230 and Cys394 is likely not required.

## Results

### Molecular modeling reveals the potential formation of disulfide bonds within the AAV capsid

**Figure 1:**
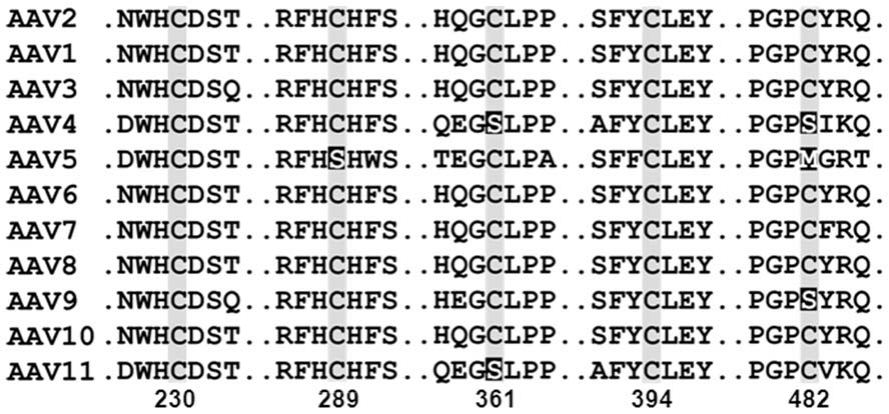
Partial sequence alignments. The relative positions of conserved cysteine residues on VP subunits of various naturally occurring AAV serotypes are shown. Numbering is based on residues within the AAV2 VP1 subunit. Alignments were obtained using Vector NTI® software.

**Figure 2:**
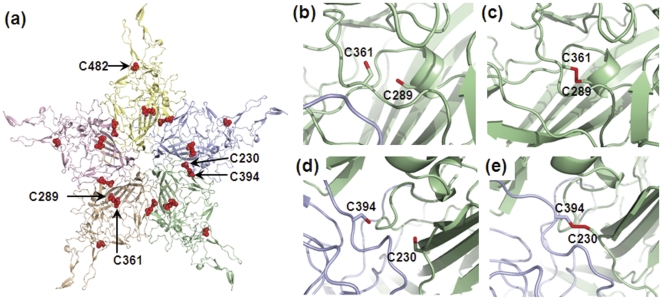
Structural analysis of cysteine residues in the AAV2 capsid VP3 subunit. (a) Locations of cysteine residues on the major capsid subunit VP3 are shown in a capsid subunit pentamer. The VP3 subunits are colored pale yellow, light blue, pale green, wheat, and light pink and the side chains of each cysteine residue are highlighted using red spheres. (b) Close up view of the positions of unpaired Cys289 and Cys361 residues within a single subunit and (d) unpaired Cys230 and Cys394 residues at the interface between two VP3 subunits at the five-fold axis of symmetry. Thiol side chains are colored red and the two VP3 subunits in pale green and light blue. Low energy snapshots from DMD simulations demonstrating the feasibility of disulfide formation between Cys289/361 pair (c) and the Cys230/394 pair (e). All images were generated using Pymol®.

The current study is focused on examining the role of cysteine residues located at five positions, i.e., 230, 289, 361, 394 and 482 (VP1 numbering) in the AAV life cycle. As shown in Figure 1, a sequence alignment comparing VP1 subunits of different AAV serotypes reveals that Cys230 and Cys394 are fully conserved. On the other hand, C289S, C361S and C482S/C482M changes are noted in AAV4, AAV5 and AAV9 suggesting that these specific cysteines might be dispensable. Molecular modeling studies using pentamer constructs of the VP3 subunit (PDB ID: 1lp3) [6] of AAV2 were carried out to locate the cysteine residues in the context of the assembled capsid. As seen in Figure 2, Cys289 and Cys361 are located adjacent to each other within a single VP subunit, while Cys230 and Cys394 are located on opposite edges of the VP subunit and appear adjacent to each other in neighboring subunits arranged along the five-fold axis of symmetry. Further, Cys482 is located within the GH loop (formed by the hypervariable region between βG and βH strands) at the base of the three-fold surface spikes as shown in the VP3 trimer (Supporting Information S1). Cartoon models of VP3 subunits with highlighted cysteine residues further show that the thiol side chain functionalities of the Cys289/361 pair and the Cys230/394 pair are separated by distances of 5.0 Å and 7.1 Å, respectively (Figure 2b & 2d).

To determine the feasibility of disulfide bond formation within each pair of cysteines, an all-atom discrete molecular dynamics (DMD) simulation of VP3 subunits was carried out within the context of a single subunit or two neighboring subunits at the five-fold symmetry axis as described in methods. Briefly, the VP3 subunit structure was maintained static, while the loops containing cysteine residues were allowed to sample possible orientations. This yielded low energy structures with disulfide bonds between the Cys289/361 (Figure 2c) as well as the Cys230/394 (Figure 2e) pairs. Thus, although no disulfide bonds have been observed in structural studies of AAV capsids until date, molecular dynamics simulations suggest that the latter are plausible.

### C289S, C361S, C482S mutants are identical to wtAAV2 virions

**Figure 3:**
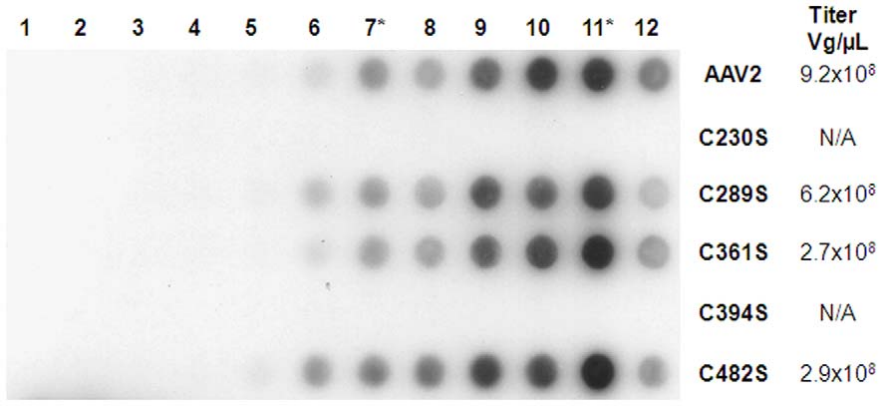
Characterization of Cys-to-Ser mutants. Western dot blot of different fractions of wtAAV2 and Cys-to-Ser mutants resolved on a cesium chloride gradient. Aliquots 7* and 11* represent peak fractions with genome-containing particles and empty virion shells, respectively. Note the absence of A20 antibody staining for C230S and C394S mutants. Vector genome titers determined by Q-PCR are indicated adjacent to the dot blot.

Based on structural cues, we generated a series of Cys-to-Ser AAV2 *Cap* mutants to interrogate the potential for disulfide bond formation (or lack thereof) in AAV capsids. Mutant and wild type AAV2 capsids packaging a firefly luciferase reporter gene were generated using the triple plasmid transfection protocol [14]. The C289S, C361S and C482S mutants assembled normally as demonstrated by a western dot blot with the A20 monoclonal antibody, which recognizes intact AAV2 capsids and yielded mutant AAV2 particles at titers comparable to that of wtAAV2 (Figure 3). Further, no significant differences in transduction efficiencies of Cys mutant AAV2 and wtAAV2 vectors in HEK293 cells were observed (Supporting Information S1). Similar transduction efficiencies were obtained in HeLa cells and upon intramuscular injection (gastrocnemius) in Balb/c mice (data not shown). Thus, mutant AAV2 capsids with C289S, C361S and C482S changes were similar to wtAAV2 capsids with regard to capsid formation, titer and transduction efficiency. Further, these results support the notion that potential disulfide bond formation between the Cys289/361 pair is nonessential.

### C230S and C394S mutants are defective in capsid formation

In contrast to the three Cys-to-Ser mutants described above, C230S and C394S mutants did not yield any genome packaging particles (Figure 3). In addition, no empty capsid particles were observed for either mutant as demonstrated by western dot blot analysis using the A20 monoclonal antibody (Figure 3). Sequencing of the entire Cys mutant AAV2 capsid (*Cap*) and replication (*Rep*) genes confirmed that no other accidental lethal mutations were present that might yield the latter phenotype. Thus, capsid protein subunits of C230S and C394S mutants do not yield infectious virions.

### C230S and C394S VP subunits are prone to proteasomal degradation

**Figure 4:**
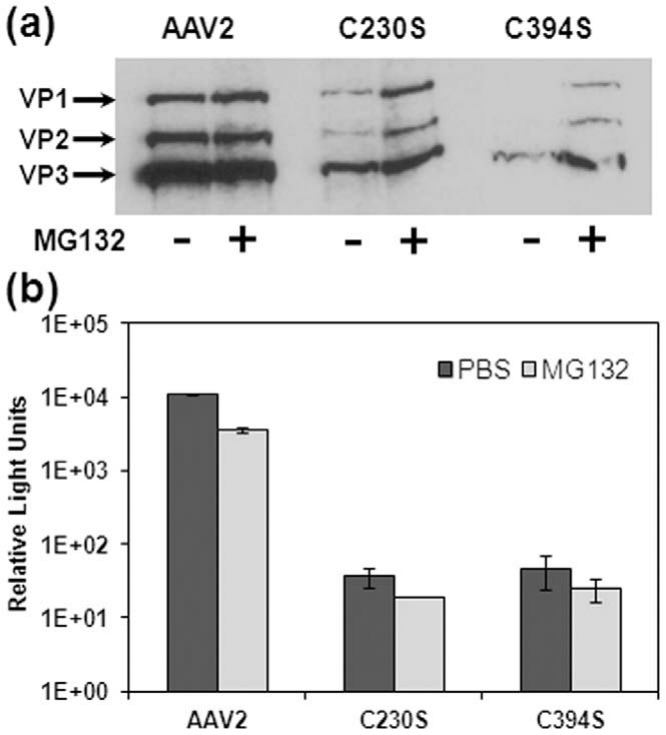
Proteasomal inhibition studies with wtAAV2, C230S and C394S mutant vectors. (a) Western blot analysis of capsid protein subunits in cell lysates using the B1 antibody at 18 hrs post-transfection with (+) or without (−) proteasome inhibitor, MG132. (b) Comparison of transduction efficiency (luciferase expression) of corresponding cell lysates shows that treatment with MG132 partially restores capsid protein expression, but not infectivity (light grey bars) when compared to control (dark grey bars). Error bars represent standard deviation (n = 3).

Expression levels of C230S/C394S mutant VP subunits were found markedly decreased when compared to wtAAV2 capsid proteins as demonstrated by western blot analysis (Figure 4a). In order to examine the likelihood that C230S/C394S mutant VP subunits were degraded by the proteasomal machinery, we co-treated 293 cells with the proteasomal inhibitor MG132 during transfection. This treatment modestly protected VP1, VP2 and VP3 subunits from proteasomal degradation (Figure 4a). However, no rescue of transducing virions was observed (Figure 4b). Taken together, the aforementioned results support the notion that mutating the Cys230 or Cys394 residue renders VP subunits prone to proteasomal degradation, which in turn could affect capsid formation.

### Further mutagenesis reveals that a Cys-to-Val substitution is tolerated at position 394, but not position 230

**Figure 5:**
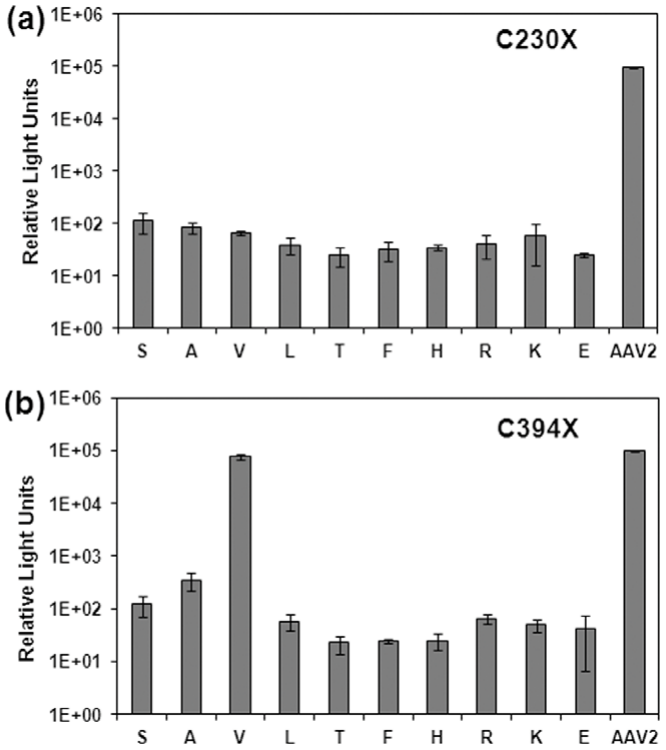
Further mutagenesis of Cys230 and Cys394 residues. Transduction efficiency (luciferase expression) of transfected cell lysates containing a panel of (a) C230 mutants and (b) C394 mutants was evaluated at 24 hr post-transduction in HEK293 cells. Only the C394V mutant displays transduction efficiency similar to wtAAV2 vectors, while the corresponding C230V mutant is not viable. Error bars represent standard deviation (n = 3).

We noted that the Cys230/394 pair is not surface exposed and located at the pentamer interface (Supporting Information S1). Interfacial residues at the icosahedral five-fold symmetry axis that surround the Cys230/394 pair include large hydrophobic residues such as tyrosine (Tyr397) and tryptophan (Trp228) (Supporting Information S1). Thus, one likely explanation is that Cys-to-Ser mutations at the 230/394 positions substantially disrupted hydrophobic interactions at the pentameric interface, thereby preventing capsid formation. Therefore, we carried out a mutagenesis study substituting a panel of hydrophobic, hydrophilic (neutral) and charged residues at positions 230/394. As seen in Figure 5a, no substitutions were tolerated at position 230 as demonstrated by the lack of infectious virions. However, a C394V mutant was viable, generating virions that transduced HEK293 cells at efficiency similar to wtAAV2 vectors (Figure 5b).

## Discussion

Cysteines are known to play critical roles in the assembly of human papillomavirus [15]–[17], portal ring formation in herpes simplex virus [18], hexameric capsomer stabilization in HIV [19] and infectivity of SV40 virions [20]–[22]. The current study is focused on investigating the role of cysteines in disulfide bond formation within AAV2 capsids. Of the five cysteines tested, VP subunits containing C230S and C394S mutations are rapidly degraded by the proteasomal machinery within the host cell. Whether these mutant VP subunits exist in monomeric or oligomeric form remains to be determined.

Interpretation of the individual role(s) played by each cysteine at the 230 or 394 position proved exceedingly difficult. The observation that a Cys-to-Val substitution can be tolerated at the 394 position, but not the 230 position suggests that disulfide bond formation by the Cys230/394 pair might be nonessential. Further, examining the secondary structure of the VP subunit reveals that Cys230 is located at the beginning of the βA strand in the VP subunit, while Cys394 is located between βE and βF strands [6]. Thus, it is plausible that mutagenesis of the Cys230 residue is more likely to disrupt secondary structure than mutations at the 394 position. It is also noteworthy to mention that mutagenesis of neighboring residues, namely, Trp228, His229 and His231 has been proposed to yield an assembly-deficient phenotype [8] (Pulicherla and Asokan, unpublished). However, this interpretation is confounded by the fact that mutations in open reading frame ORF1, which encodes capsid protein subunits also results in mutations within ORF2, which encodes the assembly-activating protein (AAP) [23]. Thus, it is likely that mutations within this region of AAP could also adversely affect AAV capsid assembly. Interestingly, complementation assays with wild type AAP are unable to rescue capsid assembly suggesting that the C230S mutant might indeed affect capsid subunit stability (Supporting Information S1). However, the possibility that mutant AAP might exert a transdominant negative phenotype cannot be ignored. Additional mutagenesis studies that might help understand interactions of Cys394 with residues in the neighboring VP subunit are difficult to carry out due to overlap between AAP and this capsid domain. Nevertheless, the latter results highlight the importance of understanding AAP-capsid protein interactions.

Why is the Cys230/394 pair unlikely to form a disulfide bond? A closer examination of the structural environment at the five-fold interface reveals that the Cys230/394 pair is nestled in a hydrophobic environment created by Phe398, Tyr397 and Trp228 residues (Supporting Information S1). The buried environment in proteins has been shown to significantly affect the pKa values and hydrogen bonding potential of cysteine thiol side chains [24]–[27]. Therefore, it is conceivable that the buried cysteines at the AAV capsid pentamer interface have high pKa values and remain protonated without forming disulfide bonds. This is further corroborated by the observation that the C394V mutation is well tolerated and does not adversely affect AAV capsid formation. Nevertheless, it is plausible that transient disulfide formation within the Cys289/361 or the Cys230/394 pair might occur in the event that conformational changes result in surface exposure of one or both cysteine thiol side chains. In summary, our results support a model wherein inter- as well as intra-subunit disulfide bond formation between neighboring cysteine residues appears nonessential for AAV capsid formation. These studies are a step towards understanding the nature of subunit interactions that drive AAV capsid assembly.

## Materials and Methods

### Molecular modeling studies

The crystal structure of the AAV2 VP3 monomer (PDB ID: 1lp3) [6] was used as a template for modeling studies. The VP3 subunit pentamer and trimer models were obtained using the online oligomer generator tool in the VIPERdb2 database [28] (http://viperdb.scripps.edu/). Cartoon models of capsid pentamers and trimers were generated and analyzed using the program Pymol (The PyMOL Molecular Graphics System, Schrödinger LLC, http://www.pymol.org/).

### Molecular simulation studies

An all-atom discrete molecular dynamics (DMD) simulations of either single VP3 subunit or two VP3 subunits within a pentamer were performed for 10^5^ time units (approximately 5 ns) [29]. To maintain the relative orientation of the monomer/dimer, the structure was maintained static and the loops containing the cysteines were allowed to sample possible orientations. Constraints were applied on the structure such that the cysteines are drawn closer in a disulfide linkage. One hundred snapshots were obtained from the simulation and the side chains of all the snapshots were repacked using Medusa [30]. The snapshot with lowest energy was considered as the final structure. The root mean square deviation of the alpha carbon atoms of the final structure with respect to the crystal structure is 1.45 Å for the loop region and 0.22 Å for the entire structure.

### Cell lines and plasmids

Human embryonic kidney (HEK) 293 cells obtained from the UNC vector core were maintained at 37°C, 5% CO_2_ in Dulbecco's modified Eagle's medium supplemented with 10% fetal bovine serum and penicillin-streptomycin-amphotericin B mixture. The AAV2 helper plasmid, pXR2 (containing AAV2 *Rep* and *Cap* genes) and pXX6-80 (containing adenoviral helper genes) were obtained from the UNC vector core. The vector cassette, pTR-CBA-Luc containing the chicken beta-actin (CBA) promoter and firefly luciferase transgene has been described earlier [31].

### Generation of AAV capsid mutants

The plasmid pXR2 was utilized as a template for site-directed mutagenesis to generate Cys-to-Ser mutations in the VP subunit region using primers (IDT, Coralville, IA) listed in Supporting Information S1. Additional mutagenesis was carried out at positions 230 and 394 using a different set of primers (Supporting Information S1). All mutagenesis reactions were carried out as per manufacturer instructions outlined in the Quikchange XL® site-directed mutagenesis kit (Agilent Technologies, Santa Clara, CA). Individual clones were sequenced at the UNC Genome Analysis facility and capsid sequences analyzed using VectorNTI® software (Invitrogen, Carlsbad, CA).

### AAV Vector production

Recombinant AAV vectors packaging a CBA promoter-driven firefly luciferase cassette were produced in HEK293 cells using the triple plasmid transfection method [14]. Transfections were carried out in 15 cm plates unless indicated otherwise. At 60 hrs post-transfection, cells were harvested and processed using cesium chloride gradient ultracentrifugation followed by dialysis. Viral titers were determined by quantitative PCR in a Roche Lightcycler® with primers (Supporting Information S1) specific for the Luc transgene region.

Transfections in 12-well plates were carried out using HEK293 cells seeded at a density of 2×10^5^ cells/well with 1 µg pXR2 (or mutant), 1.2 µg pXX6-80 and 0.6 µg pTR-CBA-Luc per well. Unless indicated otherwise, cells were harvested 24 hrs post-transfection using 250 µL deionized H_2_O and lysed by three rounds of freeze-thaw cycles (in acetone-dry ice mixture/37°C water bath) followed by treatment with DNase I. For proteasome inhibition studies, transfections were carried out in two sets of 12-well plates as described above in the presence or absence of 2 µM proteasome inhibitor MG132 (Sigma, St.Louis, MO).

### Western and dot blot analysis

Transfected cell lysates (25 µL) were subjected to SDS-PAGE and western blots carried out using the B1 monoclonal antibody which recognizes a common C-terminal epitope (IGTRYLTR) in VP subunits [32]. Dot blots (far western) of different gradient fractions were obtained using a 96-well dot blot manifold (Whatman) and probing nitrocellulose membranes with the A20 monoclonal antibody [32], which recognizes intact AAV2 capsids (conformational epitope made up of three different binding sites, VFMVPQYGYL, HYFGYSTPWG and RTTNPVATEQ).

### Transduction assays

For viral transduction assays, 5×10^4^ HEK293 cells/well were seeded in a 24-well plate format and transduced with wt or Cys mutant AAV2 vectors at MOI (multiplicity of infection) of 1000 unless indicated otherwise. At 24 hrs post-transduction, cells were lysed using 100 µL 1× passive lysis buffer (Promega, Madison, WI) and 50 µL of cell lysate transferred to 96 well assay plates for luminometric analysis in a Victor 2® luminescence plate reader (Perkin Elmer, Waltham, MA) with D-luciferin (Promega) as substrate.

## Supporting Information

Supporting Information S1 **Supporting figures and table.**
(PDF)
