# Supplementary material for: Intra- and Inter-Subunit Disulfide Bond Formation Is Nonessential in Adeno-Associated Viral Capsids
Source: PLoS One. 2012 Feb 28;7(2):e32163. doi: 10.1371/journal.pone.0032163 (PMC3289628; doi:10.1371/journal.pone.0032163)
Supplement: Supporting Information S1 — Supporting figures and table. (PDF) [file pone.0032163.s001.pdf]

SUPPORTING INFORMATION S1

Intra- and inter-subunit disulfide bond formation is nonessential in adeno-associated viral capsids

Nagesh Pulicherla[1], Pradeep Kota[3], Nikolay V. Dokholyan[3],
Aravind Asokan[1,2,3,*]

[1]Gene Therapy Center, [2]Department of Genetics, [3]Department of Biochemistry & Biophysics, The University of North Carolina at Chapel Hill, Chapel Hill, NC

Table S1. Nucleotide sequences of primers utilized in site-directed mutagenesis and Q-PCR analysis	
Name	Primer Sequence
C230S	5'- CCT CGG GAA ATT GGC ATT CCG ATT CCA CAT GGA TGG GC -3'
C289S	5'- GAC TTC AAC AGA TTC CAC TCC CAC TTT TCA CCA CGT GAC TGG -3'
C361S	5'- CGC ATC AAG GAT CCC TCC CGC CGT TCC CAG C -3'
C394S	5'- GGA CGC TCT TCA TTT TAC TCC CTG GAG TAC TTT CCT TCT C -3'
C482S	5'- TGG CTT CCT GGA CCC TCT TAC CGC CAG CAG CG -3'
C230X	5'- TCC TCG GGA AAT TGG CAT NNK GAT TCC ACA TGG ATG GGC -3'
C394X	5'- GGA CGC TCT TCA TTT TAC NNK CTG GAG TAC TTT CCT TCT C -3'
FLuc-fwd	5'- AAA AGC ACT CTG ATT GAC AAA TAC -3'
FLuc-rev	5'- CCT TCG CTT CAA AAA ATG GAA C -3'

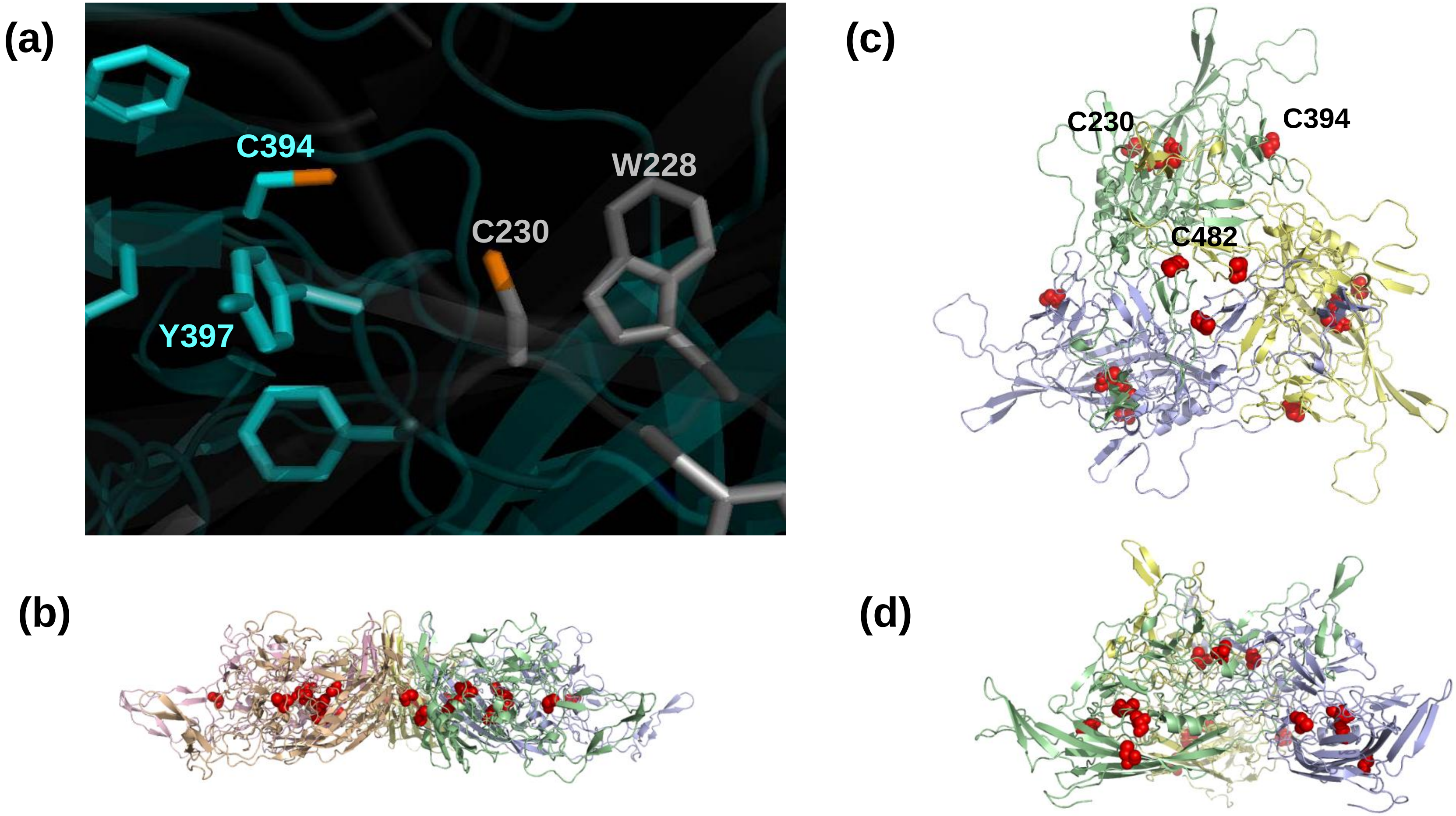

Figure S1. Structural analysis of cysteine residues in the AAV2 capsid. (a) Semi-transparent cartoon representation of two VP3 subunits at the five-fold axis of symmetry are shown colored in cyan and grey. Hydrophobic residues in close proximity W228 (grey) and Y397 (cyan) that might mediate communication between cysteines as well as stabilize the pentamer are shown. Locations of cysteine residues on the major capsid subunit VP3 are shown in (b) side view of pentamer following 90° rotation, (c) trimer and (d) side view of trimer following 90° rotation. The VP3 subunits in (b), (c), (d) are colored pale yellow, light blue, pale green, wheat, and light pink and the side chains of each cysteine residue are highlighted by red spheres. All images were generated using Pymol® (v0.99).

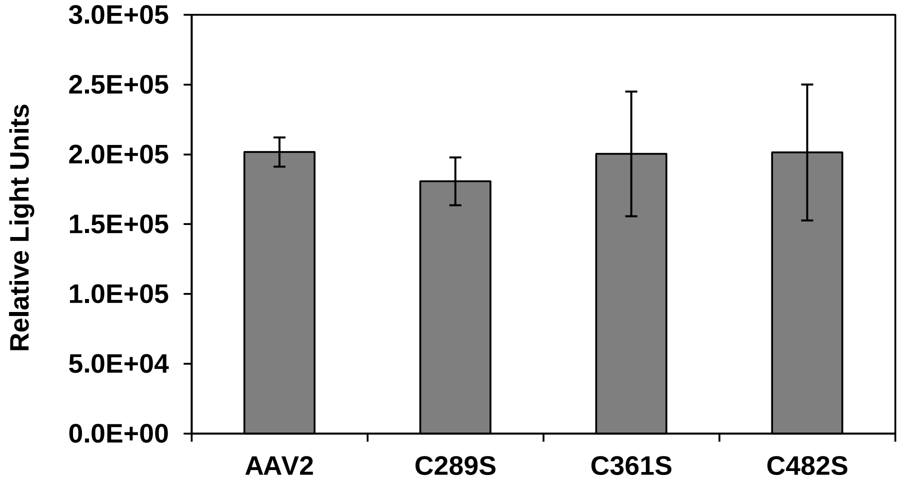

Figure S2. Transduction efficiencies (luciferase expression) of wtAAV2, C289S, C361S and C482S vectors packaging the firefly luciferase transgene in HEK293 cells (MOI = 1000). No statistically significant difference is seen. Error bars represent standard deviation (n=3).

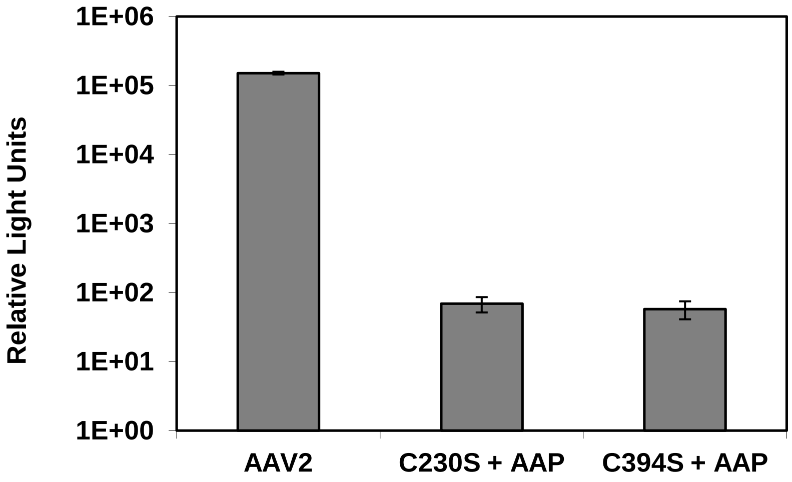

Figure S3. Capsid mixing studies with AAP plasmid (which expresses AAP alone). Transduction efficiency (luciferase transgene expression) of different cell lysates was determined at 24hr following co-transfection of mutant AAV2 plasmids and AAP plasmid at a ratio of 90:10. Neither mutant is rescued by supplementing with wild type AAP. All experiments were carried out in triplicate.
